# A population pharmacokinetics model of balovaptan to support dose selection in adult and pediatric populations

**DOI:** 10.1007/s10928-023-09898-0

**Published:** 2024-02-03

**Authors:** Franziska Schaedeli Stark, Clarisse Chavanne, Michael Derks, Koen Jolling, Hendrik Maxime Lagraauw, Lars Lindbom, Klaas Prins, Hanna E. Silber Baumann

**Affiliations:** 1 Roche Pharma Research and Early Development (pRED), Roche Innovation Center Basel, F. Hoffmann-La Roche Ltd, Grenzacherstrasse 124, 4070 Basel, Switzerland; 2 Roche Pharma Research and Early Development (pRED), Roche Innovation Center Welwyn, Roche Products Ltd, Hexagon Place, 6 Falcon Way, Welwyn Garden City, AL7 1TW UK; 3 qPharmetra LLC, Kwakkenbergweg 39, 6523MK Nijmegen, The Netherlands

**Keywords:** Balovaptan, Receptor occupancy, Pediatric dosing, Population PK, Vasopressin 1a receptor, Simulation

## Abstract

Balovaptan is a brain-penetrating vasopressin receptor 1a antagonist previously investigated for the core symptoms of autism spectrum disorder (ASD). A population pharmacokinetic (PK) model of balovaptan was developed, initially to assist clinical dosing for adult and pediatric ASD studies and subsequently for new clinical indications including malignant cerebral edema (MCE) and post-traumatic stress disorder. The final model incorporates one-compartment disposition and describes time- and dose-dependent non-linear PK through empirical drug binding and a gut extraction component with turnover. An age effect on clearance observed in children was modeled by an asymptotic function that predicts adult-equivalent exposures at 40% of the adult dose for children aged 2–4 years, 70% for 5–9 years, and at the full adult dose for ≥ 10 years. The model was adapted for intravenous (IV) balovaptan dosing and combined with in vitro and ex vivo pharmacodynamic data to simulate brain receptor occupancy as a guide for dosing in a phase II trial of MCE prophylaxis after acute ischemic stroke. A sequence of three stepped-dose daily infusions of 50, 25 and 15 mg over 30 or 60 min was predicted to achieve a target occupancy of ≥ 80% in ≥ 95% of patients over a 3-day period. This model predicts both oral and IV balovaptan exposure across a wide age range and will be a valuable tool to analyze and predict its PK in new indications and target populations, including pediatric patients.

**Supplementary Information:**

The online version contains supplementary material available at 10.1007/s10928-023-09898-0.

## Introduction

Arginine vasopressin (AVP) is a multifunctional nonapeptide hormone that mediates a range of biological activities via vasopressin 1a (V1a), 1b and 2 receptors expressed throughout the body [1]. Biological functions of AVP include the regulation of water retention [1], brain fluid homeostasis [2–4] and blood pressure [5]. AVP is synthesized primarily in the supraoptic and paraventricular nuclei of the hypothalamus, and, in addition to its peripheral functions, acts centrally as a neurotransmitter to regulate the hypothalamic-pituitary adrenocortical axis [6]. Central AVP activity has been implicated in a variety of neuropsychological functions, including the development of social behaviors and aggression [7, 8], the etiology of autism spectrum disorder (ASD) [9–15] and the onset of post-traumatic stress disorder [16]. Furthermore, V1a receptors are expressed on neurons of the suprachiasmatic nucleus that regulates circadian rhythmicity, and alterations in circadian rhythm under V1a receptor blockade [17] may potentially represent a novel treatment approach for psychiatric conditions associated with a circadian phase delay [18]. AVP has also been implicated in the onset and exacerbation of malignant cerebral edema (MCE) following acute ischemic stroke (AIS) [19–21].

Balovaptan is a brain-penetrating triazolobenzodiazepine V1a receptor antagonist [22] originally developed and investigated as an oral agent to improve social communication difficulties in adults and children with ASD [23–25]. Balovaptan is a Biopharmaceutical Classification System Class I-like molecule, with 100% oral bioavailability in adult volunteers and a systemic exposure that is not significantly affected by food [26]. It has a relatively high free drug fraction of ~ 13% in plasma [22] and is primarily metabolized by CYP3A4 [26, 27]. At steady state, balovaptan shows a consistent and roughly dose-proportional pharmacokinetic (PK) profile, with a time to oral steady state for once-daily dosing of approximately 7 days, a steady-state T_max_ of 3–4 h and a t_1/2_ of 45–47 h [26]. In contrast, following single-dose administration, non-linear PK behavior with a non-linear C_max_ and an inverse relationship between dose and t_1/2_ was observed across a similar dose range. This difference between single dose and steady state was observed across a broad dose range (0.5–76.0 mg for single dose, 12–52 mg for multiple dose) and could be explained by an inverse relationship between volume of distribution with both dose and treatment time, and also results in a dose-related non-proportionality in the balovaptan accumulation ratio calculated based on day 1 and day 14 exposure [26]. In addition, pediatric dosing in the phase II aV1ation trial in children and adolescents with ASD (NCT02901431) resulted in moderate drug underexposure at the original protocol-defined dosing [24, 28], indicating that pediatric scaling in physiologically based PK (PBPK) modeling did not adequately predict the clearance (CL)–age–weight relationship for balovaptan initial dose selection in the pediatric population.

Positron emission tomography (PET) is a molecular imaging technique used to measure the distribution of radiolabeled compounds. In neuroscience drug development it is the gold standard to quantitatively assess target or receptor occupancy (RO) of new chemical entities in the central nervous system [29]. In the absence of an available PET tracer, model-based methods in combination with in vitro and/or ex vivo experiments and receptor binding theory can be applied to achieve predictions of RO, as a surrogate for efficient target site concentration.

Although no longer being considered for ASD, balovaptan continues under clinical investigation in other indications. The objective of this work was to develop a population PK (PopPK) model of balovaptan using PK data from neurotypical individuals and adults and children with ASD. The model incorporates both non-linear exposure and age-related CL and was used to guide adult and pediatric dosing in ASD. Subsequently, the model has proven valuable for other indications. We describe herein the development and evaluation of this PopPK model, and its use to simulate brain V1a RO time courses for dosing guidance in a planned phase II trial of balovaptan for MCE prophylaxis (NCT05399550).

## Methods

### Derivation of the dataset

Data from five clinical studies were included in the PopPK analysis dataset, with 370 adult and pediatric individuals receiving balovaptan at repeated doses between 1.5 and 52 mg once-daily (QD). The majority of individuals (n = 321) were from the phase II ASD clinical trials VANILLA in adults (NCT01793441; n = 146) [23] and aV1ation in children and adolescents from 5 to 17 years of age (NCT02901431; n = 169) [24], with doses ranging from 1.5 to 10 mg QD, and relatively sparse PK sampling, mainly at steady state. These phase II study data were enriched with data from three phase I balovaptan PK studies in neurotypical adult participants, with doses ranging from 5 to 52 mg and rich PK sampling from day 1 to steady state. This combination of phase II and phase I data was considered sufficiently informative to describe and estimate non-linearity using a parsimonious approach. The studies included in the PopPK dataset are described below:

Study NCT01418963 (phase I; neurotypical adults) was a single-center, randomized, double-blind, placebo-controlled, first-in-human study to investigate the safety, tolerability and PK of balovaptan [26]. It consisted of three parts: a single-ascending dose study, a multiple-ascending dose (MAD) study and a food effect study. Only data from the 24 participants in the MAD study were included in the modeling dataset. Participants received balovaptan 12, 20, 40 or 52 mg QD (fed) in cohorts of six per dose level for a period of 14 days. Blood samples for balovaptan determination were obtained pre-dose and through 24 h post-dose on day 1, and pre-dose and through 120 h post-dose on day 14, with pre-dose samples taken on each intermediate day.

Studies NCT03579719 and NCT03586726 (both phase I; neurotypical adults) were single-center, non-randomized, open-label, one-sequence, two-period, within-subject studies with similar designs that investigated the effect of multiple-dose itraconazole and rifampicin, respectively, on the PK of multiple-dose balovaptan [27]. Only data from the control period of each study (Period 1), where balovaptan was dosed without the concomitant agent, were included in the modeling dataset. In Period 1, participants received oral balovaptan at a dose of 5 mg QD (NCT03579719; fed) or 10 mg QD (NCT03586726; fasted) for 10 days. Blood samples for balovaptan determination were obtained pre-dose at five time points up to day 10, and at fixed times up to 24 h post-dose on day 10.

Study NCT01793441 (VANILLA; phase II; adults with ASD) was a multicenter, randomized, placebo-controlled, double-blind, 12-week parallel group proof-of-concept study to investigate the efficacy and safety of balovaptan in adult men with moderate/severe ASD and an intelligence quotient (IQ) ≥ 70 [23]. Balovaptan was administered with food at doses of 1.5, 4 or 10 mg QD. Blood samples for balovaptan determination were collected pre-dose and at 2, 4 and 6 h post-dose on days 1, 14 and 84.

Study NCT02901431 (aV1ation; phase II; children and adolescents with ASD) was a multicenter, randomized, placebo-controlled, double-blind study to investigate the efficacy and safety of balovaptan in children and adolescents with ASD and an IQ ≥ 70 [24]. The study comprised an initial PK study to confirm the estimated age-adjusted balovaptan doses yielding blood systemic exposure equivalent to adult 4 mg QD and 10 mg QD, followed by a 24-week parallel two-group trial of placebo versus age-adjusted dosing equivalent to these two adult doses. Recruitment to the 4 mg QD-equivalent arm was subsequently halted early following an interim analysis, while recruitment continued into the 10 mg QD-equivalent arm. Balovaptan doses received across the various age groups over the course of both study parts were 1.5, 2, 3, 4, 5, 7 and 10 mg QD, and balovaptan was taken with or without food. Blood samples for balovaptan determination were collected in the PK cohorts at 4 h post-dose on day 1, pre-dose at day 10 in a subset of participants, and pre-dose and up to 6 h post-dose at week 2. In addition, for all patients (including the PK cohorts), samples were taken at week 8 at one post-dose time point in the evening (at home); and at weeks 12 and 24 pre-dose, 2 h post-dose and at the end of the clinic visit (≥ 4 h post-dose).

### PopPK model development

Model building was conducted in NONMEM v7.4.3 (ICON Development Solutions, Ellicott City, MD, USA) using first-order conditional estimation with interaction (FOCE-I). Data processing and post-processing of NONMEM analysis results was carried out in R v3.5.2 (Comprehensive R Network: https://cran.r-project.org/).

One- and two-compartment disposition models with first-order linear absorption and elimination were fitted to the data as a starting point for further development. Allometric scaling of CL/bioavailability (F) and apparent volume of distribution (V/F) was added a priori*,* evaluating fixed versus estimated allometric exponents, to account for the wide ranges in age and body weights, using a power relationship based on a reference weight of 76 kg. This reference weight was the median weight of the neurotypical adult volunteers in the dataset, which lay between the median weight of the adult ASD trial participants (89 kg) and the pediatric trial participants (56 kg).

Several approaches were tested to address observed non-linear PK behavior. Diagnostic plots from initial modeling steps showed a trend towards a decrease in the apparent central volume of distribution (V_c_/F) with increasing dose, indicating that saturable (i.e., capacity-limited) binding might be the underlying process. Two modeling approaches for saturable binding were explored. The first of these was the commonly used explicit binding model that describes a balovaptan mass transfer between the central and a saturable binding compartment. The second, less common approach was an empirical model that introduces a dynamic V_c_/F, as a non-linear function of the balovaptan amount in the central compartment (see Supplementary Materials for model equations). Other approaches to model non-linearity included non-linear elimination from the central compartment, non-linear bioavailability, and eventually, an empirical gut extraction component, with a hypothetical turnover compartment that controls the extent of gut extraction in an exposure- and time-dependent manner.

Absorption modeling compared a first-order absorption rate with a transit compartment absorption model (TCAM) [30], with the food effect (fed versus fasted) included in the structural model a priori. Inter-individual variability (IIV) was estimated for CL/F, V_c_/F and the absorption parameters (K_a_ or mean transit time (MTT)) using a diagonal OMEGA matrix. Several residual error models were tested to characterize the residual unexplained variability (RUV), including a proportional model, a combined additive and proportional model, and a time-varying RUV model.

Covariate analyses were undertaken for both body size and age effects on CL/F and V/F. Lean body mass (LBM) was estimated from body weight and height according to the methods described by Boer [31] for male and female adults, and by Peters et al. [32] for pediatric participants. The effect of LBM and of body mass index (BMI) were evaluated as potential alternatives to allometric scaling based on body weight. Age effects were modeled as continuous relationships using a power model or an asymptotic model.

Model selection criteria were based on the maximum likelihood of model fit (objective function value (OFV)), goodness-of-fit plots, physiological plausibility, precision of parameter estimates, model numerical stability and the condition number. The model qualification was based on acceptable visual predictive checks [33]. Confidence intervals on the parameter estimates were generated using a bootstrap approach with 500 replicates.

### Pediatric exposure simulations

The final PopPK model was used to simulate alternative age-based balovaptan dosing scenarios in a pediatric and adult population with ASD aged 2 years and above. Age–weight–sex distributions were sampled from a large database comprised of the participants from the aV1ation and VANILLA trials together with 1000 virtual participants aged 2–25 years from a pediatric and adult ASD population that was simulated in Simcyp (Certara, Inc., Princeton, NJ, USA).

Three-thousand individual PK profiles were simulated to derive steady-state area under the curve (AUC_ss_) for each dosing scenario. The target scenario was an AUC_ss_ distribution that was homogeneous across the pediatric age range and equivalent to adult exposure at all ages. Based on the results from these simulations, an updated age-based dosing algorithm was proposed for future studies of balovaptan in pediatric participants aged 2 years and above.

### IV exposure and RO simulations

Plasma kinetics and brain V1a RO following intravenous (IV) balovaptan administration were simulated to explore dosing strategies for AIS patients in Study NCT05399550. The target for optimal dosing and administration was a RO of at least 80% in at least 95% of the simulated individuals over a period of 72 h, with a subsequently rapid decline in RO thereafter.

Simulations relied on assumptions that were derived from data outside of the indicated administration route and patient group. For the simulation of plasma PK profiles it was assumed that oral CL/F and V/F were identical to IV CL and V, based on the observation of 100% bioavailability in healthy neurotypical adult individuals [26]; IV infusion was therefore modeled as a zero-order input into the central compartment with distribution and elimination unchanged from the oral model.

For the simulation of RO, balovaptan concentration at the brain V1a target site was assumed to be equivalent to the plasma-free fraction, based on negligible P-glycoprotein extraction ratio measured in vitro [22]. In absence of a suitable V1a PET tracer, balovaptan binding affinity was estimated in an ex vivo experiment using human platelet aggregates from whole blood. The results correlated well with the values from the in vitro affinity of balovaptan to the human V1a receptor and from the agonist-mediated calcium fluxes in intact cells expressing human V1a receptors using Fluorescent Imaging Plate Reader (FLIPR). Platelets are known to express the V1a receptor [34, 35] and thus the brain RO was modeled assuming that the brain receptor binding constant is the same as the platelet receptor dissociation constant. Other assumptions underlying the RO predictions include (i) PK in the (elderly) AIS population is not different from the younger population used for developing the dataset, and (ii) cerebrospinal fluid (CSF) concentration is reflective of brain distributions.

Based on these assumptions, the PK pharmacodynamic (PD) relationship for brain RO was simulated as:

$$\mathrm{RO }=\frac{{C}_{p}\times {f}_{u\_plasma}}{{K}_{b}+ \left({C}_{p}\times {f}_{u\_plasma}\right) } \times 100$$

where RO is the percent predicted brain receptor occupancy; C_p_ is the plasma balovaptan concentration; f_u_plasma_ is the plasma-free balovaptan fraction; and K_b_ is the dissociation constant.

Several dosing scenarios were simulated in order to achieve the target RO without exceeding the previously observed PK exposure. All scenarios included a high starting dose (50 or 60 mg) to achieve a rapid onset of high RO without exceeding the highest plasma concentrations observed in previous studies. For the follow-up doses, scenarios with either 24- or 36-h dosing intervals were tested, resulting in either a total of three or two doses within the 72-h treatment duration. Follow-up doses between 10 and 30 mg were evaluated. Two-thousand participants were simulated for each scenario, assuming adult “typical” parameters including the 76 kg reference weight of the neurotypical adults in the model dataset. A sensitivity analysis was also performed, assuming a body-weight distribution for a reference population of mean weight 82.2 kg with an SD of 20.3 kg and a range of 41–136 kg. These weight parameters were derived from the first 258 patients screened for entry into the ongoing TIMELESS study (NCT03785678) of tenecteplase thrombolysis immediately (4.5–24 h) after AIS (unpublished data).

## Results

### Building the PopPK model

The analysis dataset included a total of 3985 PK observations from 370 adult and pediatric individuals: 2685 observations from the phase II studies, and 1300 observations from the phase I studies. PK observations below the assay’s limit of quantitation (n = 85) had been excluded from the analysis. Demographics and balovaptan dosing information for the 370 individuals enrolled in the five included studies are shown in Table 1.

**Table 1:** Demographics and combined adult and pediatric data

	Phase I studies in neurotypical adults			Phase II studies in adults and children with ASD		Total (N = 370)
	NCT03586726 [27] (N = 16)	NCT03579719 [27] (N = 15)	NCT01418963 [26] (N = 24)	NCT01793441 [23] (N = 146)	NCT02901431 [24] (N = 169)	
Male, n (%)	16 (100.0)	12 (80.0)	21 (87.5)	146 (100.0)	145 (85.8)	340 (91.9)
Age (years), median (range)	45 (18–61)	32 (23–64)	35 (21–64)	22.5 (18–45)	14 (5–17)	19 (5–64)
Age category, n (%)						
5–7 years	0 (0.0)	0 (0.0)	0 (0.0)	0 (0.0)	11 (6.5)	11 (3.0)
8–12 years	0 (0.0)	0 (0.0)	0 (0.0)	0 (0.0)	39 (23.1)	39 (10.5)
13–14 years	0 (0.0)	0 (0.0)	0 (0.0)	0 (0.0)	66 (39.1)	66 (17.8)
15–17 years	0 (0.0)	0 (0.0)	0 (0.0)	0 (0.0)	53 (31.4)	53 (14.3)
≥ 18 years	16 (100.0)	15 (100.0)	24 (100.0)	146 (100.0)	0 (0.0)	201 (54.3)
Weight, median (range) kg	77.6 (60.2–105.0)	79.9 (50.3–99.9)	74.6 (55.1–97.5)	89.0 (51.1–152.0)	55.8 (18.8–108.0)	72.8 (18.8–152.0)
Dose received, n (%)^a^						
1.5 mg	0 (0.0)	0 (0.0)	0 (0.0)	32 (21.9)	4 (2.3)	36 (9.6)
2.0 mg	0 (0.0)	0 (0.0)	0 (0.0)	0 (0.0)	6 (3.4)	6 (1.6)
3.0 mg	0 (0.0)	0 (0.0)	0 (0.0)	0 (0.0)	35 (20.0)	35 (9.3)
4.0 mg	0 (0.0)	0 (0.0)	0 (0.0)	76 (52.1)	42 (24.0)	118 (31.4)
5.0 mg	0 (0.0)	15 (100.0)	0 (0.0)	0 (0.0)	5 (2.9)	20 (5.3)
7.0 mg	0 (0.0)	0 (0.0)	0 (0.0)	0 (0.0)	29 (16.6)	29 (7.7)
10 mg	16 (100.0)	0 (0.0)	0 (0.0)	38 (26.0)	54 (30.9)	108 (28.7)
12 mg	0 (0.0)	0 (0.0)	6 (25.0)	0 (0.0)	0 (0.0)	6 (1.6)
20 mg	0 (0.0)	0 (0.0)	6 (25.0)	0 (0.0)	0 (0.0)	6 (1.6)
40 mg	0 (0.0)	0 (0.0)	6 (25.0)	0 (0.0)	0 (0.0)	6 (1.6)
52 mg	0 (0.0)	0 (0.0)	6 (25.0)	0 (0.0)	0 (0.0)	6 (1.6)
PK sampling scheme	Pre-dose on days 2, 4, 8, 9 and 10, and 1, 2, 3, 4, 5, 6, 8, 10, 12, 16 and 24 h post-dose on days 10/11	Pre-dose on days 1, 3, 5, 8, 9 and 10, and 1, 2, 3, 4, 5, 6, 8, 10, 12, 16 and 24 h post-dose on days 10/11	Pre-dose, and 0.5, 1, 1.5, 2, 3, 4, 5, 6, 8 and 12 h post-dose on day 1; pre-dose only on days 2–14, and at 1, 2, 3, 4, 6, 8, 12, 24, 48, 96, 168 and 312 h after the final dose on day 14	Pre-dose (0 h), and 2, 4 and 6 h post-dose on days 1, 14 and 84; pre-dose (0 h) on day 42	First PK cohorts: day 1, 4 h, post-dose, day 10, pre-dose at home; All PK cohorts: week 2: pre-dose and 2, 4, and ≥ 6 h post-dose; All participants: weeks 12 and 24, pre-dose and 2 and 4 h post-dose; week 4, pre-dose, or week 8, 1 PK sample post-dose at home^b^	

^a^Six participants in pediatric trial NCT02901431 provided PK data at two different doses of balovaptan. Denominators for dose-received percentages are therefore 175 for NCT02901431 and 376 for the total dataset

^b^Some minor changes in the PK collection schedule due to protocol amendments are not reflected in this table

**Fig. 1:**
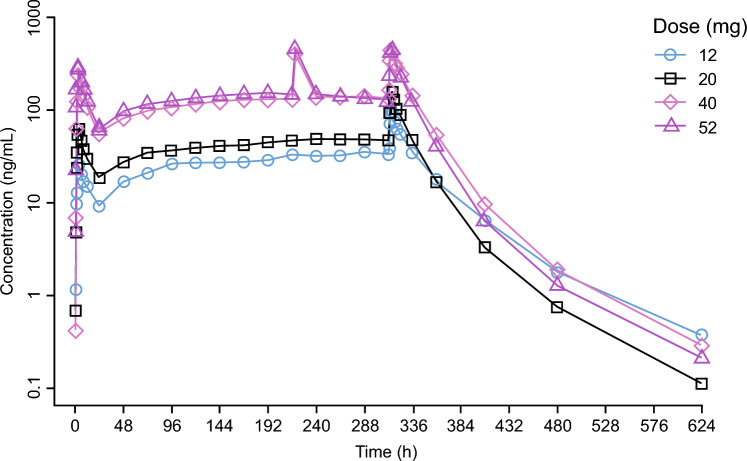
Balovaptan mean plasma PK concentrations in Study NCT01418963 after once-daily administration of 12, 20, 40 or 52 mg balovaptan for 14 days in neurotypical adult participants

**Fig. 2:**
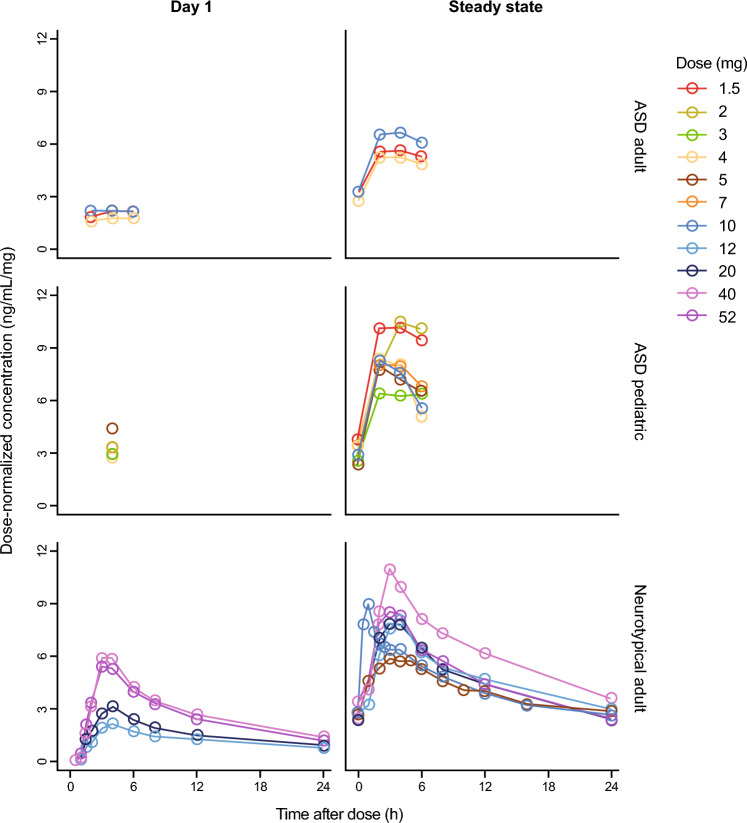
Mean dose-normalized balovaptan PK profiles after the first dose (left column) and at steady state, observed in neurotypical adults (lower row), and in adult (upper row) and pediatric (middle row) participants with ASD. Mean of at least three observed concentration values, divided by dose, are presented by nominal time points

Balovaptan PK data for the MAD part in Study NCT01418963 are presented in Fig. 1, with mean observed plasma concentrations after once-daily administration of 12, 20, 40 or 52 mg balovaptan for 14 days in neurotypical adults. Note the slightly different accumulation patterns and the more shallow terminal elimination phase for the 12 mg dose, compared with the higher doses, indicating some underlying non-linearity. Mean dose-normalized balovaptan PK profiles are presented in Fig. 2, and illustrate the different patterns observed after the first dose (day 1, left column) and at steady state, in neurotypical adults (lower row), and in adult (upper row) and pediatric (middle row) participants with ASD. The more than dose-proportional increase of exposure on day 1 becomes negligible at steady state for doses of 10 mg and above, as illustrated mainly in the lower left and right panels for neurotypical adults and is consistent with the data for the adult ASD population. Note that the 10 mg dose in Study NCT03586726 (lower right panel) was administered under fasted condition, and the earlier peak illustrates the food effect. The PK profile at 40 mg is likely confounded by a lower body weight in that subgroup. For the pediatric ASD population (middle row) the dose-normalized PK profiles are less informative since pediatric age-adjusted doses were administered in Study NCT02901431, targeting exposures equivalent to 4 mg or 10 mg doses in adults.

Starting with a linear modeling approach, a two-compartment model was supported by the PK data, with first-order absorption and elimination, and IIV on CL/F, V_c_/F and on K_a_. However, in this model individual estimates of V_c_/F were substantially correlated with dose. This non-linearity was best addressed with an empirical binding model, describing non-linear distribution, whereas non-linear elimination, describing CL/F as a function of balovaptan concentration, resulted in a less significant OFV drop. The empirical binding model was selected over the explicit binding model due to better numerical stability. However, a high condition number indicated that the empirical model needed further simplification for numerical stability. Removing the peripheral compartment resulted in full model convergence and low condition number, indicating that after including the empirical binding component, a one-compartment model described the data sufficiently well without introducing additional bias in the goodness-of-fit plots.

Absorption was best described using the TCAM approach, with fed status increasing the MTT. This is consistent with previous work demonstrating the superiority of the TCAM approach over many first-order absorption models featuring a lag time [30], particularly in situations with a high degree of inter- and intraindividual variability, as was evidenced by this dataset.

**Fig. 3:**
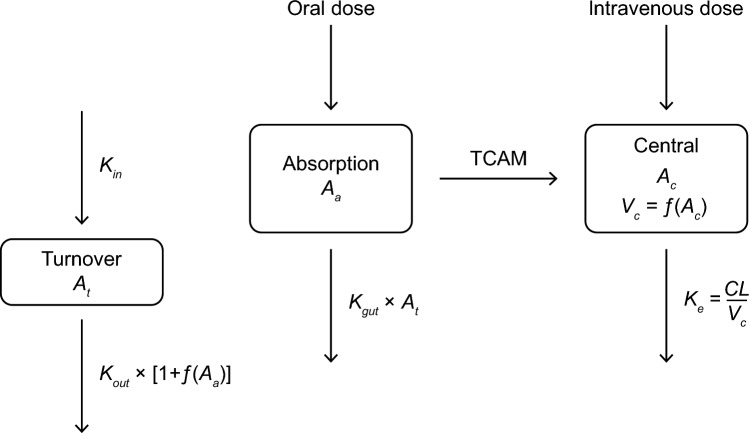
Illustration of the final PopPK model. The oral drug is dosed into the absorption compartment (A_a_) from where it is absorbed with a delay (TCAM) into the central compartment (A_c_) and eliminated with a first-order process (K_e_). A dynamic central volume of distribution (V_c_) reflects an empirical approach to describe capacity-limited binding. V_c_ decreases as a function of the amount of drug in the central compartment (A_c_), reaching its minimum when the available (hypothetical) binding sites are saturated. This process is modeled by the empirical function: ${V}_{c}={V}_{0}\times \left(1-{VL}_{max}\times \frac{ {A}_{c}}{{A}_{c} + {VL}_{A50}}\right)$. A gut elimination process from the absorption compartment is controlled by a hypothetical turnover compartment (A_t_), that can assume values between 1 (when A_a_ = 0) and 0 (when A_a_ is high), with a time delay controlled by the values of K_in_ and K_out_. Thus, gut elimination depends on the dose amount as well as on the dose interval for repeated doses

A remaining bias in conditional weighted residuals versus time was eliminated through the incorporation of a gut extraction process from the absorption compartment. The gut extraction process is a time- and dose-dependent process, modeled as an empirical turnover compartment (A_t_), with initial value of 1, and first-order input and output rate constants K_in_ and K_out_. The value of A_t_ is controlling the gut extraction rate constant (K_gut_), while the amount in the absorption compartment (A_a_) reduces A_t_ through stimulation of K_out_, thereby introducing a time delay between the increase of amount in the absorption compartment and the decrease of K_gut_ (see Fig. 3). Thus, with increasing amount in the absorption compartment, through increasing dose level or increasing number of repeated doses, gut extraction decreases to insignificant levels.

Following inclusion of the empirical binding model and the gut extraction model, there was no remaining bias in the goodness-of-fit displays, indicating that the model adequately captured the PK disposition properties. A food effect was observed in the data and needed to be incorporated in the model for an adequate description of the absorption time course. However, only 4.3% of the participants with PK data were fasted, and the food effect parameter could not be precisely estimated, which resulted in a high condition number. The food effect on MTT was therefore fixed in the model to an estimate of 3.39 h [26], which substantially improved the condition number. In addition, fixing the gut extraction degradation rate K_out_ further improved the numerical stability of the model. Residual variability was high immediately after balovaptan administration, and lower after a few hours post-dose. A time-varying RUV model was introduced, with a mono-exponential decay from a high value immediately after dosing to a lower level after the absorption phase, with a half-life of 0.53 h. This time-varying proportional RUV, together with an additive RUV fixed to a value equal to half the lower limit of quantitation, significantly improved the model fit and stabilized convergence.

Scaling of V_c_/F was directly proportional to total body weight and fixed as such, while the estimated power for weight on CL/F was low (0.256) although statistically significant. Substitution with BMI or LBM as alternative body size measures for scaling CL/F and V/F resulted in worse fits and were rejected. The relationship between CL/F and body weight appeared to be stronger in the pediatric population than in adults. Therefore, an empirical maturation function for CL/F with age was tested, using a non-linear asymptotic function describing pediatric CL/F as a fraction of adult CL/F. This model improved the model fit, and the effect of weight on CL/F was no longer significant.

### Obtaining the final PopPK model structure and parameters

The final model was a one-compartment structure featuring a TCAM absorption component and a gut extraction model, with linear elimination from the central compartment and an empirical binding model that expresses V_c_/F as a function of balovaptan concentration in the central compartment (Fig. 3). This model is mathematically described by the equations:

$$\frac{d{A}_{a}}{dt}=Input-{K}_{tr}\times {A}_{a}-{A}_{t}\times {K}_{gut}\times {A}_{a}$$

$$\frac{d{A}_{c}}{dt}={K}_{tr}\times {A}_{a}-{K}_{e}\times {A}_{c}$$

$$\frac{d{A}_{t}}{dt}={K}_{in}-{K}_{out}\times {A}_{t}\times \left(1+S\times {A}_{a}\right)$$

With the initial condition of A_t_ set to 1, and S is the scaling factor for the effect of A_a_ on the gut extraction degradation rate K_out_. The micro-constants of mass transfer were defined as:

$${K}_{in}={K}_{out}$$

$${K}_{tr}=\frac{{N}_{tr}+1}{MTT}$$

$${K}_{e}=\frac{CL}{{V}_{c}}$$

The central volume of distribution was defined as a function of the amount in the central compartment:

$${V}_{c} = {V}_{0} \times \left(1- {VL}_{max} \times \frac{{A}_{c}}{ {A}_{c}+ {VL}_{A50}}\right)$$

The covariate relationship of body weight (WT) on V_c_/F was defined by a power model, and that of age on CL/F by an asymptotic function:

$${CL}_{i}=CL\times \left(1-\frac{1}{{e}^{{-AGE}_{slope}}\times \left({AGE}_{50}-AGE\right)}\right)$$

$${V}_{c,i}={V}_{c}\times \left(\frac{WT}{76}\right)$$

The final model parameter estimates are shown in Table 2.

**Table 2:** Parameter estimates of the final PopPK model

Parameter	Description	Estimate	95% CI
CL (L/h)	Apparent clearance	8.52	7.70–9.64
V_0_ (L)	Apparent volume of distribution at baseline	565	468–686
MTT (h)	Mean transit time	0.367	0.34–0.39
VL_max_ (fraction)	Maximum fraction of volume decrease	0.805	0.771–0.839
VL_A50_ (μg)	Amount in the central compartment resulting in 50% of the maximum volume decrease	3.36	2.16–5.21
N_tr_	Number of transit compartments	5.81	4.29–7.26
K_gut_ (1/h)	Gut extraction rate constant	1.89	1.16–2.88
K_out_ (1/h)	Gut extraction degradation rate constant	0.00205	(Fixed)
S	Scaling factor for the effect of A_a_ on K_out_	22.0	13.8–30.5
WT_V0_	Effect of body weight on V_0_	1.00	(Fixed)
FOOD_MTT_	Effect of food on MTT	3.39	(Fixed)
RUV_early_	Maximum value of time-varying RUV immediately after dosing	1.20	0.811–1.77
RUV_late_	Minimum value of time-varying RUV	0.216	0.201֪–0.232
RUV_rate_ (1/h)	RUV decrease rate	1.32	0.992–1.670
RUV_add_	Additive RUV component	0.000625^a^	(Fixed)
AGE_slope_	Slope in the CL–age maturation function	0.267	0.106–0.447
AGE_50_ (years)	Age where 50% of adult CL/F is reached	5.34	1.36–6.89
IIV_CL_	Variance on CL/F	0.150^b^	0.120–0.177
IIV_V0_	Variance on V_0_	0.0944^b^	0.0628–0.1320
IIV_MTT_	Variance on MTT	0.0423^b^	0.0232–0.0661

95% CI calculated from a 500-sample bootstrap, with 457 successful minimizations

The time-varying (proportional) RUV model was implemented as $RU{V}_{prop}=RU{V}_{early}-(RU{V}_{early}-RU{V}_{late})\times (1-{e}^{{RUV}_{rate} \times TAD})$, where RUV_prop_ is the proportional RUV component and TAD is time after dosing

^a^The additive RUV component was fixed to a value equivalent to 0.025 ng/mL standard deviation, corresponding to 50% of the assay lower limit of quantitation

^b^IIV values are reported as variance estimates in the log-normal distribution. Coefficient of variation was 39% for IIV_CL_, 31% for IIV_V0_ and 21% for IIV_MTT_. Shrinkage was 4.5% for IIV_CL_, 17.4% for IIV_V0_, and 40.8% for IIV_MTT_

**Fig. 4:**
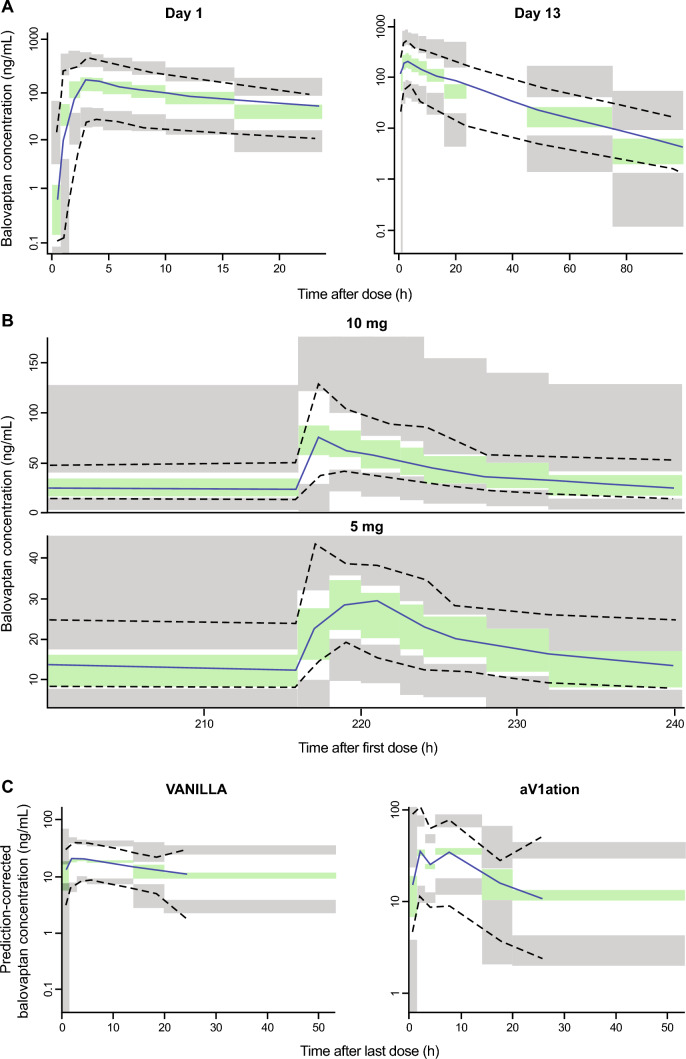
Visual predictive checks of the final PopPK model stratified by study. **A** NCT01418963; **B** NCT03586726 (10 mg) and NCT03579719 (5 mg); **C** NCT01793441 (VANILLA) and NCT02901431 (aV1ation). Median, 5th and 95th percentiles of observed data (solid and dashed lines) are overlain to the simulated 95% confidence intervals (shaded areas). **A** VPC versus time since last dose for Study NCT01418963 day 1 (left panel) and day 13 (right panel); **B** VPC versus time since first dose for the steady-state PK profiles in NCT03586726 (10 mg, upper panel) and NCT03579719 (5 mg, lower panel); **C** prediction-corrected VPC versus time since last dose for the steady-state PK profiles in NCT01793441 (VANILLA, left panel) and NCT02901431 (aV1ation, right panel)

Visual predictive checks (Fig. 4) and goodness-of-fit (Supplementary Figs. 1 and 2) demonstrated appropriate agreement between predicted and observed data values. Distributions of empirical Bayesian estimates plotted versus covariate values (dose, formulation, study, race, sex, age, various body size measures) indicated an appropriate and unbiased model fit (not shown).

**Fig. 5:**
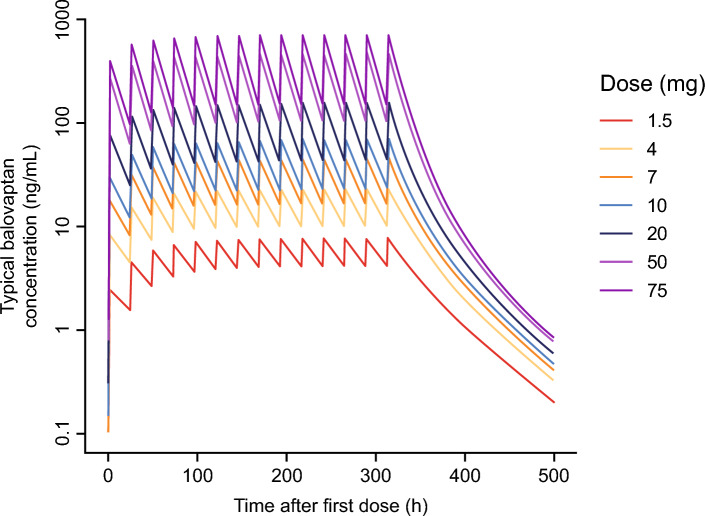
Simulations of balovaptan PK profiles for a typical individual: 1.5 to 75 mg once-daily for 14 days

**Fig. 6:**
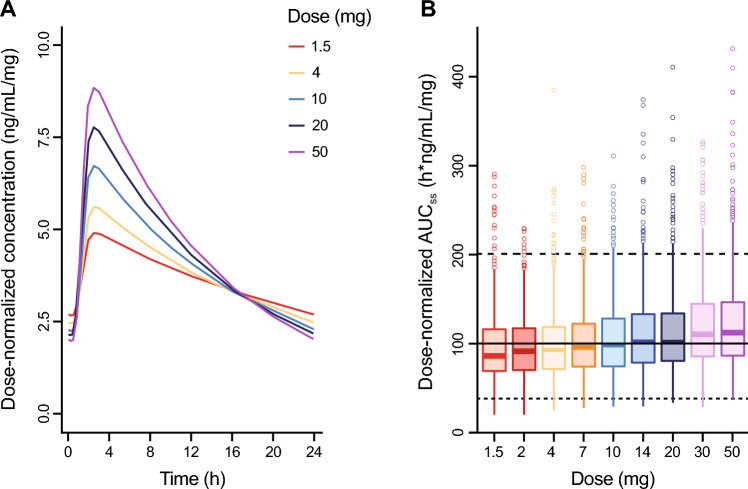
Simulations of balovaptan dose-normalized exposure at steady state for 1.5 to 50.0 mg once-daily dosing. **A** Predicted dose-normalized PK profiles for a typical adult participant. **B** Distribution of simulated dose-normalized AUC_ss_ by dose for a neurotypical population (N = 1000). Solid and dashed horizontal lines indicate the dose-normalized median, and 5th and 95th percentiles of observed AUC_ss_ in an adult ASD population in VANILLA

Simulations were performed to explore the effects of the non-linearity terms in the model on the adult oral balovaptan PK profile at day 1 and at steady state (Figs. 5 and 6).

### Effect of age on balovaptan PK and updated pediatric dosing

**Fig. 7:**
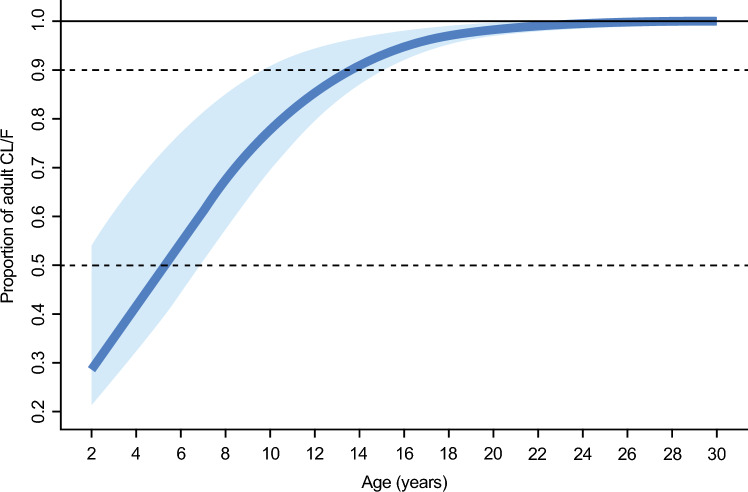
Effect of age on apparent clearance. Solid line shows proportion of adult mean CL/F modeled by an asymptotic function (see text) based on population mean parameter estimates AGE_slope_ (0.267) and AGE_50_ (5.35 years; age at 50% of adult clearance). Shaded area represents uncertainty in the age–clearance relationship derived from the 95% CI around AGE_50_ (1.4–6.9 years). Dashed lines represent 50% and 90% of adult CL/F

Under the asymptotic model retained in the final PopPK model, predicted balovaptan CL/F increases steeply with age to a plateau at approximately 20 years, with ~ 90% of adult CL/F achieved at age 14 years (Fig. 7).

### Balovaptan brain RO kinetics under IV dosing

**Fig. 8:**
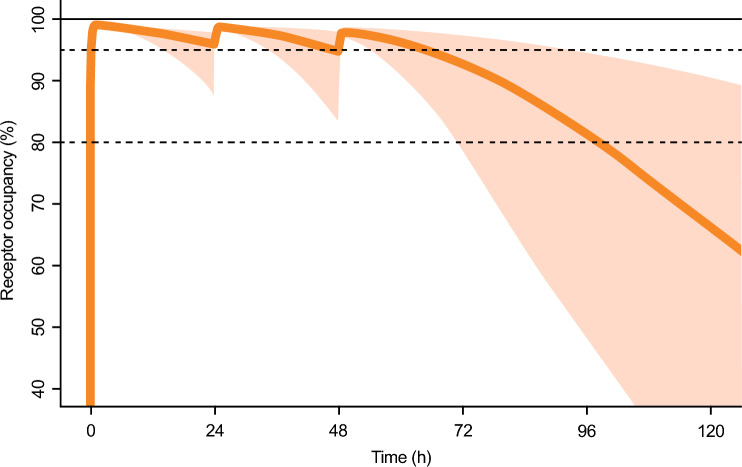
Simulated median brain receptor occupancy kinetics and 90% prediction interval following sequential daily intravenous infusions (60 min) of balovaptan 50 mg, 25 mg, and 15 mg. Solid line: median; shaded area: 90% prediction interval from 2000 simulated participants. Horizontal dashed lines indicate 80% and 95% receptor occupancy

The initial simulations indicated that a starting dose of 50 mg given as a 60-min infusion resulted in rapid and near-complete V1a RO within 30 min. The RO following the initial dose remained above the defined target limit for 24, but not 36 h, and therefore a three-dose strategy with dosing every 24 h was needed. The second and third dose (at 24 and 48 h) were selected to be as low as possible to meet the defined target limit and at the same time enable a rapid reduction of the RO after 72 h. The dosing regimen that was closest to meeting these criteria consisted of three doses (50, 25 and 15 mg), administered with a 24-h interval, and is illustrated in Fig. 8. This dosing regimen largely fulfills the criteria for RO with the exception that the RO falls below the limit just before reaching the 72-h mark, which was considered acceptable.

Table 3 summarizes simulated plasma concentrations and brain RO at discrete time points under the same schedule, along with data from the sensitivity analysis using the heavier patient weights derived from the TIMELESS study. The predictions for the sensitivity analysis can be seen to be closely similar to those using the “typical” model parameters, including the size of the slight dip of the 5th percentile below 80% RO at 72 h.

**Table 3:** Simulated blood concentrations and brain V1a receptor occupancies over 5 days following three 60-min IV infusions of balovaptan 50, 25, and 15 mg on days 1, 2, and 3

	Day 1		Day 2		Day 3		Day 5
	1 h	24 h	25 h	48 h	49 h	72 h	96 h
Blood concentration (ng/mL), median and 5th to 95th percentile range							
Ref 76 kg	352 (215–566)	74 (23–147)	261 (176–392)	59 (17–149)	166 (107–268)	42 (12–117)	15 (3–57)
Ref 82.2 ± 20.3 kg	332 (175–624)	74 (22–144)	254 (161–406)	61 (16–145)	167 (1–2–270)	43 (12–118)	15 (3–63)
Brain receptor occupancy (%), median and 5th to 95th percentile range							
Ref 76 kg	99 (98–99)	96 (88–98)	99 (98–99)	95 (83–98)	98 (97–99)	93 (79–97)	82 (48–95)
Ref 82.2 ± 20.3 kg	99 (98–99)	96 (87–98)	99 (98–99)	95 (83–98)	98 (97–99)	93 (78–97)	82 (46–95)

Data at 24 h and 48 h above are pre-infusion; data at 1, 25 and 49 h are immediately post-infusion

## Discussion

We have described the development of a PopPK model for balovaptan that is characterized by one-compartment disposition with delayed oral absorption described by a TCAM. Time- and dose-dependent non-linearity in the balovaptan PK profile was addressed by two model features: an empirical binding model of balovaptan that expresses the volume of distribution as a decreasing function of the amount of balovaptan in the central compartment, and a gut extraction process with a turnover compartment that scales the extraction rate as an inverse function of the amount of balovaptan in the absorption compartment. The main sources of data were two phase II studies in adult and pediatric participants (aV1ation and VANILLA). Additional phase I data resulted in a dataset with doses in a range of 5 to 52 mg and with rich PK sampling both on day 1 and at steady state, which allowed the dose and time non-linearities in balovaptan PK to be fully addressed.

Simulations were performed to explore the effects of the non-linearity terms in the model on the adult oral balovaptan PK profile at day 1 and at steady state. As seen in Fig. 5, across a dose range of 1.5–75.0 mg QD, lower doses had a longer predicted time to steady state and greater systemic accumulation, and the elimination phase was exposure-dependent, which is consistent with the dose and time effects on balovaptan PK noted in empirical studies [26]. Figure 6A illustrates how the non-linearity terms in the model translate into predicted dose effects at steady state, with a greater than dose-proportional increase in C_max_ and decrease in C_min_ with increasing dose resulting from the non-linear volume of distribution introduced by the empirical binding model. Further, the derived dose-normalized AUC_ss_ estimates illustrated in Fig. 6B show that despite these kinetic effects on maximum and minimum drug concentrations, non-linearity has a limited effect on steady-state overall exposure. The gut extraction component in the model leads to slightly more than dose-proportional AUC_ss_, but median systemic availability across the dosing range of 1.5 mg to 50.0 mg QD is expected to remain within − 10% and + 15% of the reference adult dose of 10 mg used in clinical studies of ASD. A mechanistic explanation for this gut extraction component in the model is currently lacking; however, the hypothetical turnover compartment, which was inspired by commonly used models for enzyme induction or inhibition, was suitable to address and quantify the remaining dose- and time-dependent effect observed in the data, and exposure predictions within the analyzed dose range are expected to be reliable.

The model therefore exemplifies an empirical, albeit simplified, representation of capacity-limited binding in describing a non-linear volume of distribution [36]. It is of interest that an explicit binding model, the most common approach to address this type of behavior, was unstable in the modeling process, while the far less common approach of modeling a dynamically variable volume of distribution via empirical balovaptan binding worked well. The cause of the non-linearity, which was first observed in early first-in-human and bioavailability studies of balovaptan [26], is unknown, but would be consistent with saturable binding to peripheral V1a receptors such as on platelets [35]. These early balovaptan data are consistent with the empirical binding model in two significant ways: firstly, in that the observed pattern of non-linearity is consistent with the dynamic volume of distribution introduced by the model, and secondly in that the empirical model predicts an exposure-dependent elimination half-life (and hence time to steady state) that is consistent with the observed data in these earlier studies. Of relevance to any such peripheral receptor binding, it should be noted that no obvious safety signals have been observed to date in children or adults receiving balovaptan in phase I–III clinical and pharmacology studies [23–27], and no clinically relevant effects on cardiology, heart rate or blood pressure were noted among neurotypical adults receiving balovaptan 10 or 50 mg QD for 2 weeks in a cardiac safety study (NCT03808298) [37].

The asymptotic function describing the age-related difference in pediatric versus adult clearance rates predicts approximate adult CL/F at the age of 14 years and above. No significant age-related effect is predicted in adults up to the age of 65 years. Simulations from the final model resulted in an updated dosing recommendation for pediatric populations aged 2 to 17 years, compared with the dosing algorithm used in the aV1ation study (in which the final age-adjusted dosing was to give adult doses to children aged 8 years and above, and 70% of the adult dose to those aged 5–7 years). The updated recommendation was to give adult doses for children aged 10 years or above, 70% of the adult dose for children aged 5–9 years and 40% of the adult dose for children aged 2–4 years. These data are consistent with observations from the aV1ation trial that showed moderate under-dosing only among children under 15 years. The original protocol-defined dosing for children was derived using physiology-based PK modeling, which assumed a larger age-related effect on balovaptan clearance compared with what was later identified [24, 28]. The majority of these children (91% [105/116] in the PK dataset) were aged between 8 and 14 years, whose clearance values would have varied between 70 and 90% of that of adults under the final age model.

Of note, scaling by age and body size (body weight or lean/fat-free body weight) is often considered the gold standard to describe pediatric CL and volume of distribution, relative to adult CL [38]. For children aged 2 years and above, age-based maturation usually approaches adult levels and body-size scaling has been discussed as optimal for dose adjustment. In our analysis, however, body-weight scaling was initially included in the model, and weight-based allometric scaling of the volume of distribution was supported by the data. However, once an empirical maturation function on CL/F was included in the model, weight-based scaling of CL was no longer significant, which led to the conclusion that body-size correction on the clearance estimate is required for the pediatric, but not for the adult populations. Thus, although age and body size are closely correlated in the pediatric population, our model indicates that age-based pediatric dose adjustment is appropriate and can be used to guide balovaptan dosing to provide adult-equivalent exposures in children aged 2 years and above. This finding is in line with recent work by Cleary et al. [39] who demonstrated higher enzyme activity in children aged 1–10 years, requiring an age-based maturation function to adequately describe pediatric PK of risdiplam. Furthermore, Upreti et al. [40] have reported higher CYP4A4 enzyme activity for children up to 10 years of age than predicted based on in vitro ontogeny data.

Another feature of interest for the model is the simulation of lower oral bioavailability at low balovaptan doses, which is a function of the gut extraction process extracting a greater proportion of the dose when the amount of balovaptan in the absorption compartment is low. This results in a small dose dependency for steady-state AUC, as well as larger dose dependencies for steady-state C_max_ and C_min_ attributable to volume of distribution effects introduced by the empirical balovaptan binding model. These effects result in AUC_ss_ distributions at very low and very high doses within about 10–15% of the 10 mg QD reference, which is not considered to be of clinical significance.

Given the essentially complete oral bioavailability of balovaptan at adult dosing levels, the model lends itself readily to adaptation for IV administration, and simulations of IV PK profiles were in good agreement with oral dosing trajectories after allowing for the oral absorption delay. The flexibility of the model for oral or IV administration is a valuable feature as balovaptan continues in clinical development for indications requiring both approaches. The concentration–time profile following a 30–60 min IV infusion was found to overlap well with the concentration–time profile following oral administration once a 15-min absorption lag time had been accounted for [26], which further supported the validity of using the model to predict PK following IV administration.

One important initial requirement for the model development was that it would provide a detailed simulation of pediatric oral drug exposures to allow definitive recommendations for adult-equivalent pediatric dosing in future balovaptan studies. Another important requirement was that, in combination with in vitro and ex vivo PD data, it was able to simulate IV PK–PD profiles for brain V1a RO, and to guide infusion schedules for the NCT05399550 phase II study of MCE prophylaxis. The model successfully achieved both these requirements, firstly providing both a three-stratum dose adjustment schedule for very young (2–4 years), young (5–9 years) and older children (10 + years) and secondly, establishing a sequential stepped-dose infusion schedule to maintain brain V1a RO over a critical 3-day period following AIS. The PK–PD modeling process successfully identified both suitable and unsuitable dosing and administration schedules for achieving the target RO and established that three sequential daily infusions of 50 mg, then 25 mg and finally 15 mg would maintain > 80% target brain RO for the 3 days, with a rapid subsequent decline. The selected target RO was supported by evolving evidence indicating that high RO levels of up to 90% are required for effective inhibition of G-protein coupled receptors [41].

It should be noted that several caveats apply. The PD modeling process necessarily incorporated reasonable—but currently untestable—assumptions that brain tissue penetration would mirror observed CSF penetration, and that the kinetics of brain RO would mirror observed binding to peripheral receptors. Similarly, there is also an implicit assumption that brain penetration and PD activity is not significantly affected by AIS, and that stroke patients, many of whom are elderly, will necessarily demonstrate the same balovaptan PK profiles as the generally much younger dataset from whom the PopPK model was derived.

In conclusion, we have developed a model of balovaptan PopPK that successfully incorporates both time- and dose-dependent non-linearity and age-associated clearance variability. This model is a valuable tool for analyzing and predicting PK data for balovaptan in other indications and target populations, and for guiding pediatric dosing to achieve adult-equivalent systemic exposures.

### Supplementary Information

Below is the link to the electronic supplementary material.

Supplementary file1 (PDF 912 kb)

## References

[1] Birnbaumer M (2000). Vasopressin receptors. Trends Endocrinol Metab.

[2] Hertz L, Chen Y, Spatz M (2000). Involvement of non-neuronal brain cells in AVP-mediated regulation of water space at the cellular, organ, and whole-body level. J Neurosci Res.

[3] Niermann H, Amiry-Moghaddam M, Holthoff K, Witte OW, Ottersen OP (2001). A novel role of vasopressin in the brain: modulation of activity-dependent water flux in the neocortex. J Neurosci.

[4] Raichle ME, Grubb RL (1978). Regulation of brain water permeability by centrally-released vasopressin. Brain Res.

[5] Koshimizu TA, Nasa Y, Tanoue A, Oikawa R, Kawahara Y, Kiyono Y, Adachi T, Tanaka T, Kuwaki T, Mori T, Takeo S, Okamura H, Tsujimoto G (2006). V1a vasopressin receptors maintain normal blood pressure by regulating circulating blood volume and baroreflex sensitivity. Proc Natl Acad Sci USA.

[6] Engelmann M, Landgraf R, Wotjak CT (2004). The hypothalamic-neurohypophysial system regulates the hypothalamic-pituitary-adrenal axis under stress: an old concept revisited. Front Neuroendocrinol.

[7] Hammock EA, Young LJ (2006). Oxytocin, vasopressin and pair bonding: implications for autism. Philos Trans R Soc Lond B Biol Sci.

[8] Insel TR (2010). The challenge of translation in social neuroscience: a review of oxytocin, vasopressin, and affiliative behavior. Neuron.

[9] Kim SJ, Young LJ, Gonen D, Veenstra-VanderWeele J, Courchesne R, Courchesne E, Lord C, Leventhal BL, Cook EH, Insel TR (2002). Transmission disequilibrium testing of arginine vasopressin receptor 1A (AVPR1A) polymorphisms in autism. Mol Psychiatry.

[10] Parker KJ, Oztan O, Libove RA, Mohsin N, Karhson DS, Sumiyoshi RD, Summers JE, Hinman KE, Motonaga KS, Phillips JM, Carson DS, Fung LK, Garner JP, Hardan AY (2019). A randomized placebo-controlled pilot trial shows that intranasal vasopressin improves social deficits in children with autism. Sci Transl Med.

[11] Tansey KE, Hill MJ, Cochrane LE, Gill M, Anney RJ, Gallagher L (2011). Functionality of promoter microsatellites of arginine vasopressin receptor 1A (AVPR1A): implications for autism. Mol Autism.

[12] Wassink TH, Piven J, Vieland VJ, Pietila J, Goedken RJ, Folstein SE, Sheffield VC (2004). Examination of AVPR1a as an autism susceptibility gene. Mol Psychiatry.

[13] Yang SY, Cho SC, Yoo HJ, Cho IH, Park M, Kim BN, Kim JW, Shin MS, Park TW, Son JW, Chung US, Kim HW, Yang YH, Kang JO, Kim SA (2010). Association study between single nucleotide polymorphisms in promoter region of AVPR1A and Korean autism spectrum disorders. Neurosci Lett.

[14] Yang SY, Kim SA, Hur GM, Park M, Park JE, Yoo HJ (2017). Replicative genetic association study between functional polymorphisms in AVPR1A and social behavior scales of autism spectrum disorder in the Korean population. Mol Autism.

[15] Yirmiya N, Rosenberg C, Levi S, Salomon S, Shulman C, Nemanov L, Dina C, Ebstein RP (2006). Association between the arginine vasopressin 1a receptor (AVPR1a) gene and autism in a family-based study: mediation by socialization skills. Mol Psychiatry.

[16] Sipos E, Torok B, Barna I, Engelmann M, Zelena D (2020). Vasopressin and post-traumatic stress disorder. Stress.

[17] Yamaguchi Y (2018). Arginine vasopressin signaling in the suprachiasmatic nucleus on the resilience of circadian clock to jet lag. Neurosci Res.

[18] Vidafar P, Yocum AK, Han P, McInnis MG, Burgess HJ (2021). Late chronotype predicts more depressive symptoms in bipolar disorder over a 5 year follow-up period. Int J Bipolar Disord.

[19] Ameli PA, Ameli NJ, Gubernick DM, Ansari S, Mohan S, Satriotomo I, Buckley AK, Maxwell CW, Nayak VH, Shushrutha Hedna V (2014). Role of vasopressin and its antagonism in stroke related edema. J Neurosci Res.

[20] Dickinson LD, Betz AL (1992). Attenuated development of ischemic brain edema in vasopressin-deficient rats. J Cereb Blood Flow Metab.

[21] Zeynalov E, Jones SM, Elliott JP (2020). Vasopressin and vasopressin receptors in brain edema. Vitam Horm.

[22] Schnider P, Bissantz C, Bruns A, Dolente C, Goetschi E, Jakob-Roetne R, Kunnecke B, Mueggler T, Muster W, Parrott N, Pinard E, Ratni H, Risterucci C, Rogers-Evans M, von Kienlin M, Grundschober C (2020). Discovery of balovaptan, a vasopressin 1a receptor antagonist for the treatment of autism spectrum disorder. J Med Chem.

[23] Bolognani F, Del Valle RM, Squassante L, Wandel C, Derks M, Murtagh L, Sevigny J, Khwaja O, Umbricht D, Fontoura P (2019). A phase 2 clinical trial of a vasopressin V1a receptor antagonist shows improved adaptive behaviors in men with autism spectrum disorder. Sci Transl Med.

[24] Hollander E, Jacob S, Jou R, McNamara N, Sikich L, Tobe R, Smith J, Sanders K, Squassante L, Murtagh L, Gleissl T, Wandel C, Veenstra-VanderWeele J (2022). Balovaptan vs placebo for social communication in childhood autism spectrum disorder: a randomized clinical trial. JAMA Psychiat.

[25] Jacob S, Veenstra-VanderWeele J, Murphy D, McCracken J, Smith J, Sanders K, Meyenberg C, Wiese T, Deol-Bhullar G, Wandel C, Ashford E, Anagnostou E (2022). Efficacy and safety of balovaptan for socialisation and communication difficulties in autistic adults in North America and Europe: a phase 3, randomised, placebo-controlled trial. Lancet Psychiatry.

[26] Derks M, Lennon-Chrimes S, Guenther A, Squassante L, Wandel C, Szczesny P, Paehler A, Kletzl H (2021). Bioavailability and pharmacokinetic profile of balovaptan, a selective, brain-penetrant vasopressin 1a receptor antagonist, in healthy volunteers. Expert Opin Investig Drugs.

[27] Derks MGM, Wandel C, Young A, Bolt SK, Meyenberg C (2020). Open-Label assessment of the effects of itraconazole and rifampicin on balovaptan pharmacokinetics in healthy volunteers. Adv Ther.

[28] Schaedeli Stark F, Chavanne C, Lennon-Chrimes S, Diack C, Derks M, Smith J (2020). P.133 Paediatric dosing of balovaptan for the treatment of the core symptoms of autism spectrum disorder: data from a phase 2 study (aV1ation; NCT02901431). Eur Neuropsychopharmacol.

[29] Varrone A, Bundgaard C, Bang-Andersen B (2022). PET as a translational tool in drug development for neuroscience compounds. Clin Pharm Therap.

[30] Savic RM, Jonker DM, Kerbusch T, Karlsson MO (2007). Implementation of a transit compartment model for describing drug absorption in pharmacokinetic studies. J Pharmacokinet Pharmacodyn.

[31] Boer P (1984). Estimated lean body mass as an index for normalization of body fluid volumes in humans. Am J Physiol.

[32] Peters AM, Snelling HLR, Glass DM, Bird NJ (2011). Estimation of lean body mass in children. Br J Anaesth.

[33] Bergstrand M, Hooker AC, Wallin JE, Karlsson MO (2011). Prediction-corrected visual predictive checks for diagnosing nonlinear mixed-effects models. AAPS J.

[34] Thibonnier M, Roberts JM (1985). Characterization of human platelet vasopressin receptors. J Clin Invest.

[35] Inaba K, Umeda Y, Yamane Y, Urakami M, Inada M (1988). Characterization of human platelet vasopressin receptor and the relation between vasopressin-induced platelet aggregation and vasopressin binding to platelets. Clin Endocrinol.

[36] Smith DA, Van Waterschoot RA, Parrott NJ, Olivares-Morales A, Lavé T, Rowland M (2018). Importance of target-mediated drug disposition for small molecules. Drug Discov Today.

[37] Derks M, Wandel C, Jordan P, Scoon S, Young A, Bolt S (2020) No clinically relevant effect of balovaptan on electrocardiography, heart rate or blood pressure in healthy volunteers (abstract 416.003). In: Proceedings and abstracts of the international society for autism research virtual meeting 2020 (June 3). pp 309–310. https://insar.confex.com/insar/f/noxeeosbroze. Accessed Nov 2022

[38] Anderson BJ, Holford NH (2011). Tips and traps analyzing pediatric PK data. Paediatr Anaesth.

[39] Cleary Y, Kletzl H, Grimsey P, Heinig K, Ogungbenro K, Silber Baumann HE, Frey N, Aarons L, Galetin A, Gertz M (2023). Estimation of FMO3 ontogeny by mechanistic population pharmacokinetic modelling of risdiplam and its impact on drug–drug interactions in children. Clin Pharmacokinet.

[40] Upreti VV, Wahlstrom JL (2016). Meta-analysis of hepatic cytochrome P450 ontogeny to underwrite the prediction of pediatric pharmacokinetics using physiologically based pharmacokinetic modeling. J Clin Pharmacol.

[41] Grimwood S, Hartig PR (2009). Target site occupancy: emerging generalizations from clinical and preclinical studies. Pharmacol Ther.

