# Supplementary material for: A population pharmacokinetics model of balovaptan to support dose selection in adult and pediatric populations
Source: J Pharmacokinet Pharmacodyn. 2024 Feb 3;51(3):227–42. doi: 10.1007/s10928-023-09898-0 (PMC11136808; doi:10.1007/s10928-023-09898-0)
Supplement: Supplementary file 1 — Supplementary file1 (PDF 912 kb) [file 10928_2023_9898_MOESM1_ESM.pdf]

A population pharmacokinetics model of balovaptan to support dose selection in adult and pediatric populations

Journal of Pharmacokinetics and Pharmacodynamics

Franziska Schaedeli Stark[1] · Clarisse Chavanne[1] · Michael Derks[2] · Koen Jolling[3]

Hendrik Maxime Lagraauw[3] · Lars Lindbom[3] · Klaas Prins[3] · Hanna E. Silber Baumann[1]

[1]Roche Pharma Research & Early Development (pRED), Roche Innovation Center Basel, F. Hoffmann-La Roche Ltd, Grenzacherstrasse 124, 4070 Basel, Switzerland

[2]Roche Pharma Research & Early Development (pRED), Roche Innovation Center Welwyn, Roche Products Ltd, Hexagon Place, 6 Falcon Way, Welwyn Garden City, AL7 1TW, UK

[3]qPharmetra LLC, Kwakkenbergweg 39, 6523MK Nijmegen, The Netherlands

Corresponding author:

Franziska Schaedeli Stark, PhD

Email: franziska.schaedeli_stark@roche.com

Supplementary Figure 1 Observed data versus final PopPK model individual and population predictions: (A) linear plot; (B) log plot. Solid line, Loess smooth; dashed line, line of identity

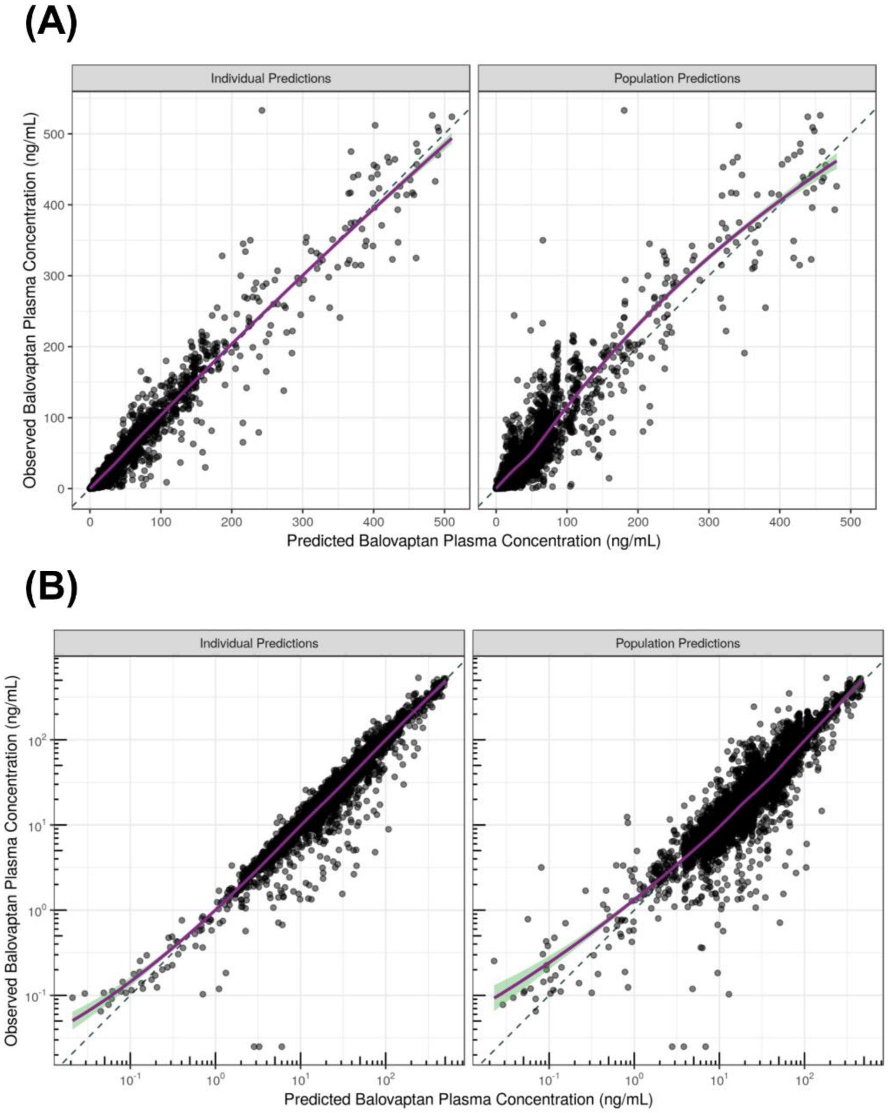

Supplementary Figure 2 Final PopPK model conditional weighted residual (CWRES) assessments by individual study. (A) CWRES versus time after first dose; (B) CWRES versus time after first dose at steady state; (C) CWRES versus time after last dose; (A–C solid line, Loess smooth; dashed line, zero); (D) Estimated CWRES versus theoretical quantiles of the normal distribution (solid line through 25th and 75th percentiles); (E) Density plot and histogram of CWRES (solid line, density of N[0,1])

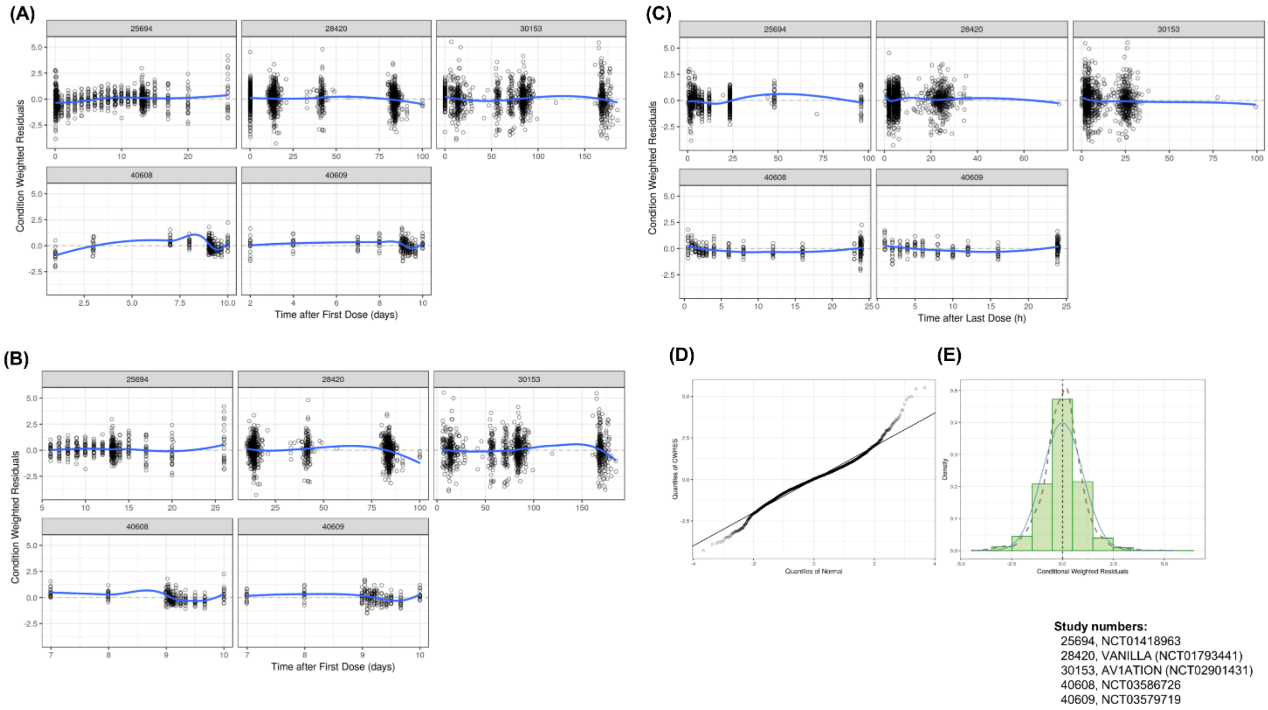

Supplementary material: empirical versus explicit binding models

The explicit binding model (below), which included a saturable rate constant describing the transport of balovaptan to the peripheral binding compartment, significantly improved OFV (ΔOFV –660), but did not converge and was unstable during estimation:

$$\frac{dA_a}{dt} = -K_a \times A_a$$

$$\frac{dA_c}{dt} = K_a \times A_a - \frac{B_{max} \times K_{pc} \times A_c}{K_m + A_c} + K_{pc} \times A_p - K_e \times A_c$$

$$\frac{dA_p}{dt} = \frac{B_{max} \times K_{pc} \times A_c}{K_m + A_c} - K_{pc} \times A_p$$

Where the elimination rate constant was defined as $K_e = \frac{CL}{V_c}$, subscript "a" refers to absorption, "c" to the central compartment, and "p" to the peripheral compartment.

By contrast, an empirical binding model (below) that described distribution volume as a decreasing E_{max} function based on the amount of balovaptan in the central compartment, resulted in a larger drop in OFV (ΔOFV –887) and removed the bias between V_c/F and dose:

$$\frac{dA_a}{dt} = -K_a \times A_a$$

$$\frac{dA_c}{dt} = K_a \times A_a - K_{cp} \times A_c + K_{pc} \times A_p - K_e \times A_c$$

$$\frac{dA_p}{dt} = K_{cp} \times A_c - K_{pc} \times A_p$$

Where the central volume of distribution was defined as: $V_c = V_0 \times \left(1 - \frac{VL_{max} \times A_c}{A_c + VL_{A50}}\right)$

And the rate constants were defined as:

$$K_e = \frac{CL}{V_c}$$

$$K_{cp} = \frac{Q}{V_c}$$

$$K_{pc} = \frac{Q}{V_p}$$
